# Force-plate derived predictors of lateral jump performance in NCAA Division-I men’s basketball players

**DOI:** 10.1371/journal.pone.0284883

**Published:** 2023-04-21

**Authors:** Charles R. Reiter, Carolyn Killelea, Mallory S. Faherty, Ryan J. Zerega, Caroline Westwood, Timothy C. Sell

**Affiliations:** 1 Virginia Commonwealth University School of Medicine, Richmond, Virginia, United States of America; 2 Michael W. Krzyzewski Human Performance Laboratory, Orthopaedic Surgery, Duke University School of Medicine, Durham, North Carolina, United States of America; 3 Department of Orthopaedic Surgery, Duke University Medical Center, Durham, North Carolina, United States of America; 4 OhioHealth Research Institute, OhioHealth, Columbus, Ohio, United States of America; 5 Atrium Health Musculoskeletal Institute, Charlotte, North Carolina, United States of America; 6 Lewis Katz School of Medicine at Temple University, Philadelphia, Pennsylvania, United States of America; Kennedy Krieger Institute/Johns Hopkins University School of Medicine, UNITED STATES

## Abstract

A lateral jump assessment may provide unique benefits in sports such as basketball that require multidirectional performance optimization. This study aimed to examine selected force-plate derived metrics as predictors of lateral jump task distance in men’s basketball players. Twenty-two NCAA Division-I men’s basketball players (19.4 ± 1.3 years, 95.0 ± 12.5 kg, 196.5 ± 8.1 cm) each performed six single leg lateral jumps while standing on a force plate (1200 Hz, Kistler Instrument Corp). The lateral jump task involved the subject beginning by standing on the force plate and jumping sideways off one foot and then landing on the floor with the opposite foot. Three-dimensional ground reaction force curves were used to identify the eccentric and concentric phases of the jump and variables were computed each from the lateral (y), vertical (z), and resultant (r) force traces. Peak ground reaction force (pGRF), ground reaction force angle (θ_r_), eccentric braking rate of force development (ECC-RFD), average concentric force (CON-AVG), total jump duration, eccentric phase duration, and eccentric to total time ratio were evaluated for predictive ability. Three regression models were able to significantly (p<0.05) predict jump distance: (1) pGRF_y_, pGRF_z_, and θ_r_ (p<0.001, R^2^ = 0.273), (2) Relative pGRF_y_, Relative pGRF_z_, and θ_r_ ((p<0.001, R^2^ = 0.214), and (3) Relative CON-AVG_y_ and Relative pGRF_r_ (p<0.001, R^2^ = 0.552). While several force plate-derived metrics were identified as significant predictors, a model with Relative CON-AVG_y_ and Relative pGRF_r_ explained a greater variability in performance (R^2^ = 0.55) compared to the other variables which were low, yet also significant. These results suggest that lateral ground reaction forces can be used to evaluate lateral jump performance with the use of three-dimensional force plates. The identified predictors can be used as a starting point for performance monitoring, as basketball training interventions can be directed at specific improvements in the identified metrics.

## Introduction

Jump assessments, such the countermovement jump (CMJ) [1–18], are frequently used to quantify an athlete’s lower extremity neuromuscular ability. These assessments have shown benefits for evaluating sport-specific performance [1, 4, 6–8, 16, 18–22], assessing neuromuscular readiness and fatigue [2, 3], and guiding return-to-sport recommendations [23]. In basketball, jumps are one of the core tasks central to the game and performing powerful and explosive lower body movements are crucial both offensively and defensively [24]. Additionally, lower extremity injuries account for approximately two-thirds of all men’s and women’s collegiate basketball injuries [25, 26]. Therefore, a basketball-specific battery of jump tasks and other assessments may help guide improvements in both performance and injury prevention and recovery.

Although jump assessments are often exclusively vertical, a lateral jump assessment may be a beneficial inclusion in musculoskeletal evaluations for basketball players. CMJs are typically vertical because the unidirectional and controlled motion allows for the simple derivation of instantaneous velocity and displacement from the force-time signature using force plate outputs [10]. Velocity and displacement can then be used to distinguish the eccentric and concentric phases of the jump motion [2–4, 6, 11, 13, 14], both of which represent a fundamental component of the muscular stretch shortening cycle (SSC). Many different metrics can be derived from the displacement and velocity curves and they allow for evaluation of specific components of an athlete’s SSC [11, 27]. Metrics that have been identified as predictors of task performance can then be targeted for specialized modifications. Predictors of vertical CMJ performance have been determined previously (e.g., average concentric force and eccentric braking rate of force development) and can serve as a point of emphasis for optimal training [6, 28].

The vertical CMJ’s ability to evaluate multidirectional movement performance, however, may be limited [12]. Basketball involves many multidirectional high and moderate intensity movements [29] and 20% of these movements occur laterally [30]. Thus, a lateral assessment may thus provide information that a vertical assessment cannot. Lateral assessments have not been studied to the same extent as vertical assessments, likely due to the greater cost and lesser portability of three-dimensional force plates. The identification of force plate derived predictors for lateral jump performance may allow for individualized lateral performance optimization as training interventions can be targeted for specific aspects of the lateral SSC; however, strong predictors have yet to be determined [13].

The purpose of this study was to examine force plate derived predictors of lateral jump performance in male NCAA Division-I basketball players. Potential predictors included both kinetic (peak ground reaction force, average concentric force, and eccentric braking rate of force development) and kinematic (jump duration, eccentric phase duration, and eccentric to total duration ratio) metrics. Various potential predictor variables were measured using three-dimensional force plates and computed from each of the lateral, vertical, and resultant ground reaction force output curves. It was hypothesized that a multiple regression model based on these variables would be able to significantly predict lateral jump distance. Further, it was predicted that the model would include lateral kinetic metrics, in addition to vertical kinetic and kinematic metrics. These findings hope to identify areas of focus for lateral performance improvement in elite basketball players, as well as highlight a potential benefit of quantifying lateral ground reaction forces with three-dimensional force plates.

## Materials and methods

### Experimental approach to the problem

Collegiate men’s basketball players underwent a lateral jump task off a three-dimensional force plate. To identify predictors of lateral jump performance, variables that had been previously used to predict vertical CMJ performance [6, 27] were computed from the force-time curves of the force plate outputs during the task and ultimately correlated to achieved lateral distance. Metrics included peak ground reaction force, average concentric force, and eccentric braking rate of force development, jump duration, eccentric phase duration, and eccentric to total duration ratio. Each metric was computed from each of three ground reaction force vectors–lateral, vertical, and two-dimensional resultant–to fully quantify the multidirectional neuromuscular actions undergone during a lateral movement. Variables with statistically significant correlations were incorporated into a prediction model for jump distance. A statistically significant model could be used to highlight key neuromuscular qualities that can be modified to optimize lateral performance.

### Subjects

Twenty-two NCAA Division-I men’s basketball players participated in the study. All participants provided written informed consent approved by Duke University’s Institutional Review Board (No. Pro00084165). Testing was conducted during the summer prior to the start of each basketball season from 2017 to 2019. Participants had a mean age of 19.4 ± 1.3 years, body mass of 95.0 ± 12.5 kg, and height of 196.5 ± 8.1 cm at the time of testing. All subjects were free of injury and medically cleared for basketball activities.

### Procedures

#### Lateral jump task

**Fig 1:**
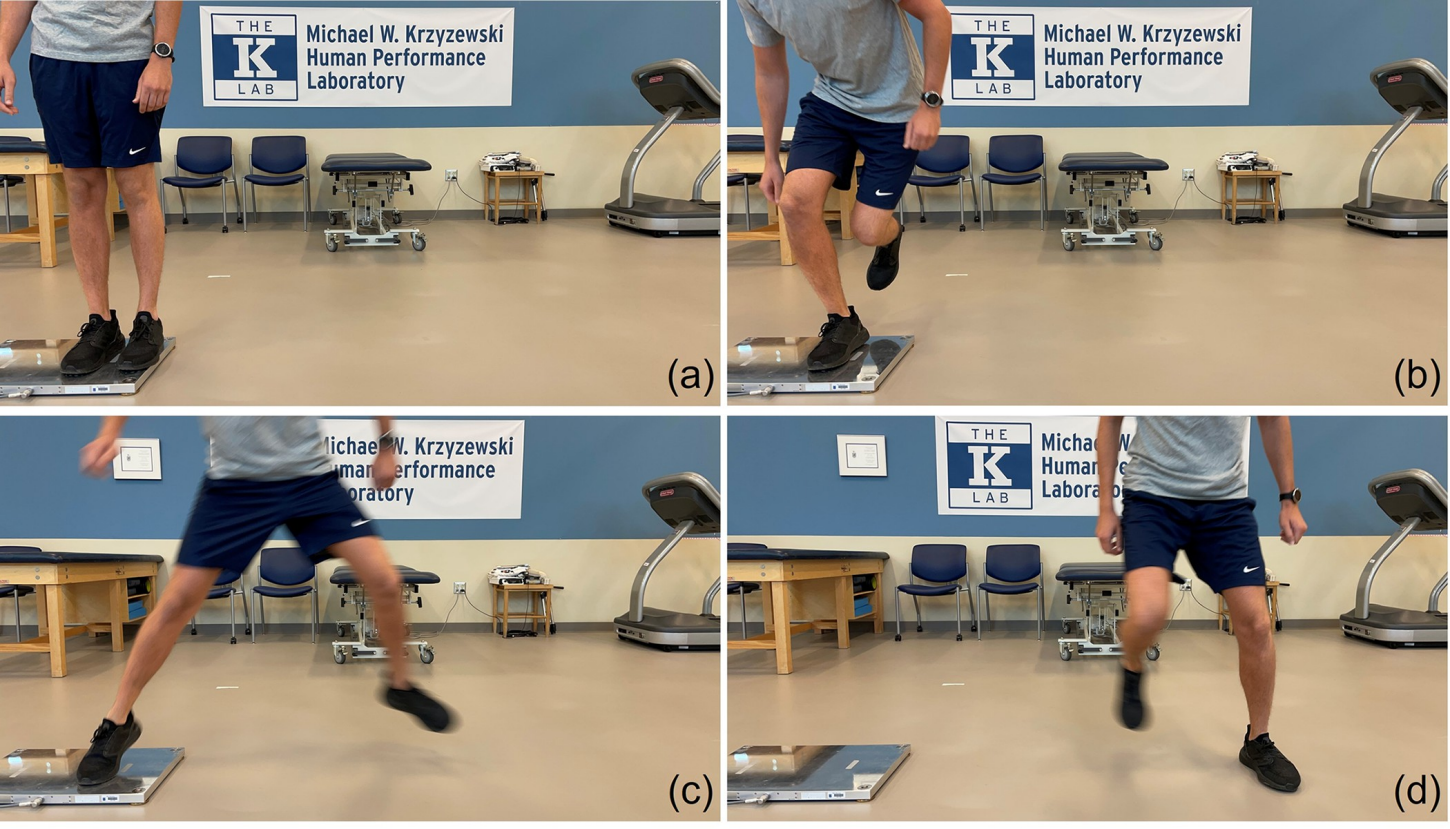
Lateral jump task protocol. Images represent (a) start point on force plate, (b) lift opposite leg, (c) takeoff on planted leg, (d) land on opposite leg.

A strength and conditioning coach led all participants in a warmup routine before testing that included dynamic, multidirectional movements and dynamic stretching. The task consisted of the subject jumping sideways off one foot and landing on the opposite foot. Subjects were instructed to stand on the force plate, lift the opposite leg, and jump sideways as far as possible (Fig 1). Arm swings were permitted. Participants were allowed one jump to practice the movement. The procedures were repeated for a total of six acceptable trials—three jumping off the right leg and three off the left leg. An acceptable trial was one in which the subject took off on one foot and held the landing on the opposite foot. An unacceptable trial was one in which the subject did not takeoff on only one foot, landed on both feet instead of one, or shifted their foot on landing. Subjects were given approximately 30 seconds of rest time between jumps.

The lateral jump distance was measured with a tape measure from the medial edge of the takeoff foot to the medial edge of the landing foot to the nearest 0.01 m. Three-dimensional ground reaction forces were recorded during the takeoff phase at a sampling rate of 1200 Hz using one portable Kistler force plate (9286BA; Kistler Instrument Corp, Novi, MI) and Vicon Nexus Software (version 2.6.1, Vicon, Centennial, CO). Force data was collected until a vertical force threshold below 5% body weight (BW) was reached, signifying takeoff [31, 32].

### Data reduction

Data reduction and analysis was conducted on a per trial basis for a total of 200 trials. There were two instances of a subject only performing the task on one side and four instances of recording only two acceptable trials for a given leg due to time restraints. This accounts for 10 missing trials and 4.8% missing data.

Raw force plate data was filtered with a 4^th^ order zero lag Butterworth filter with a cutoff frequency of 100 Hz [33]. The filtered three-dimensional ground reaction force data was exported to MATLAB for further analysis. In MATLAB, three force-time curves were derived: vertical ground reaction force (F_z_), lateral ground reaction force (F_y_), and vertical-lateral resultant ground reaction force (F_r_). The vertical-lateral resultant force curve was computed as the resultant magnitude of the vertical and lateral force curves. The MATLAB script was then used to identify critical time points in the force curves and compute various potential predictor variables.

The F_z_ curve was used to identify the time point of jump initiation and of takeoff. Jump initiation was defined as the point at which the F_z_ dropped and remained at least 5% below body weight (BW) for the during of the unloading phase [34, 35]. Takeoff was defined as the point at which F_z_ fell below 5% BW, thus indicating the subject was no longer in contact with the force plate [31, 32].

The vertical, lateral, and resultant peak and relative peak ground reaction force (pGRF) and resultant ground reaction force angle at the time of peak resultant force (θ_r_) were computed for the full set of jump trials. The pGRF was computed as the maximum force in Newtons recorded during the jump motion for each ground reaction force curve. The peak relative ground reaction force (N/kg) was computed by normalizing the peak force to body mass in kilograms. θ_r_ was computed at the instance of pGRF_r_ by finding the inverse tangent of F_z_ divided by F_y_. It should be noted that θ_r_ is not a trajectory or takeoff angle as F_z_ includes the contribution of gravity. This variable was intended to detail the directionality of F_r_ as measured by the force plate and was why the contribution of gravity was not removed.

**Fig 2:**
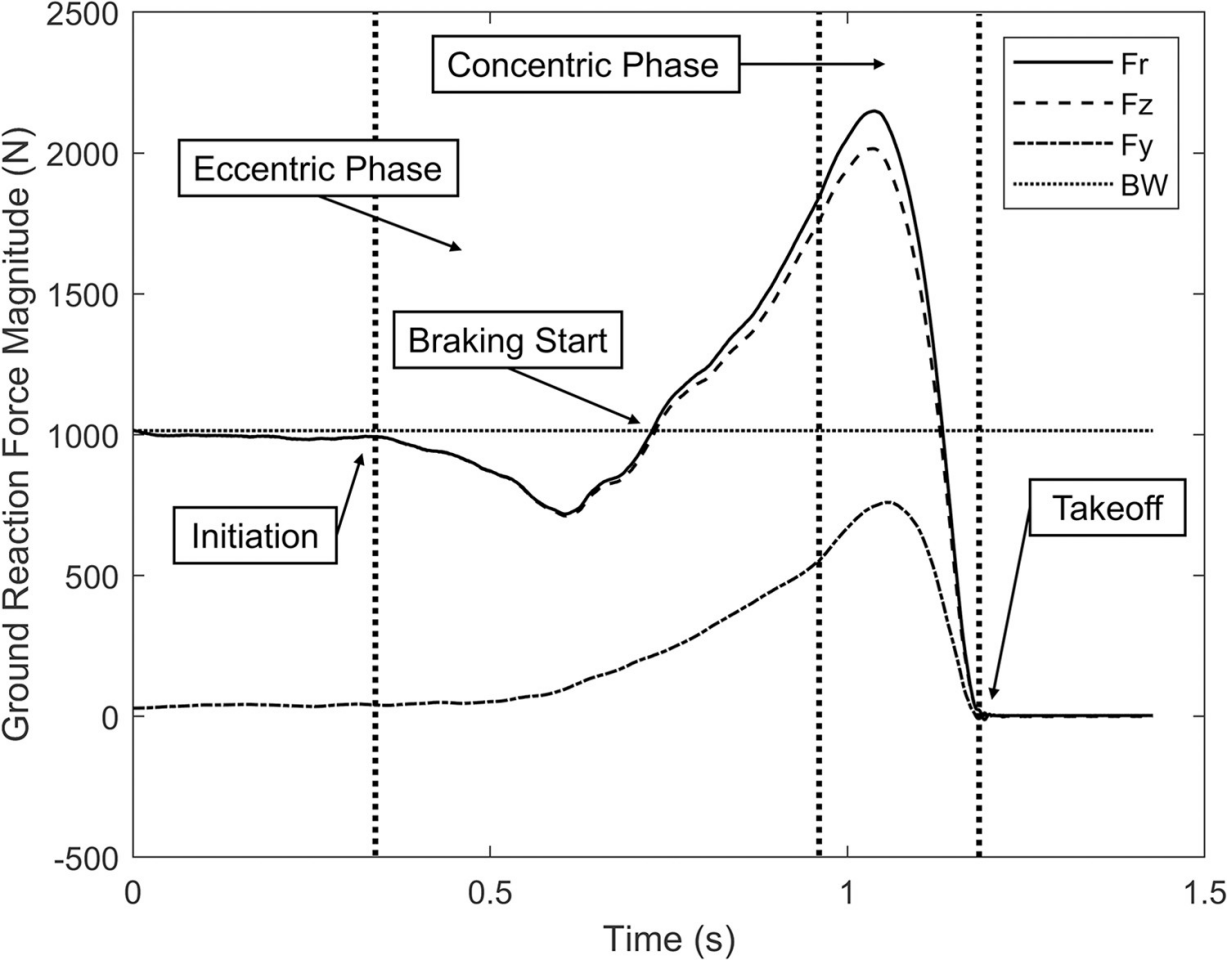
Example force vector plot. Phase boundaries noted.

The eccentric and concentric phases of the lateral jump motion were identified to compute additional metrics. A velocity-time curve was derived from the vertical force-time curve by integration using the trapezoidal rule and was used to identify phase boundaries. The eccentric phase was defined as the point of jump initiation to the point of minimum vertical displacement or zero vertical velocity. The eccentric braking phase begins at the point in which vertical force exceeds body weight and ends at the beginning of the concentric phase. The concentric phase was defined as the end of the eccentric phase to takeoff [10]. An example jump trial with relevant boundaries noted can be seen in Fig 2.

**Fig 3:**
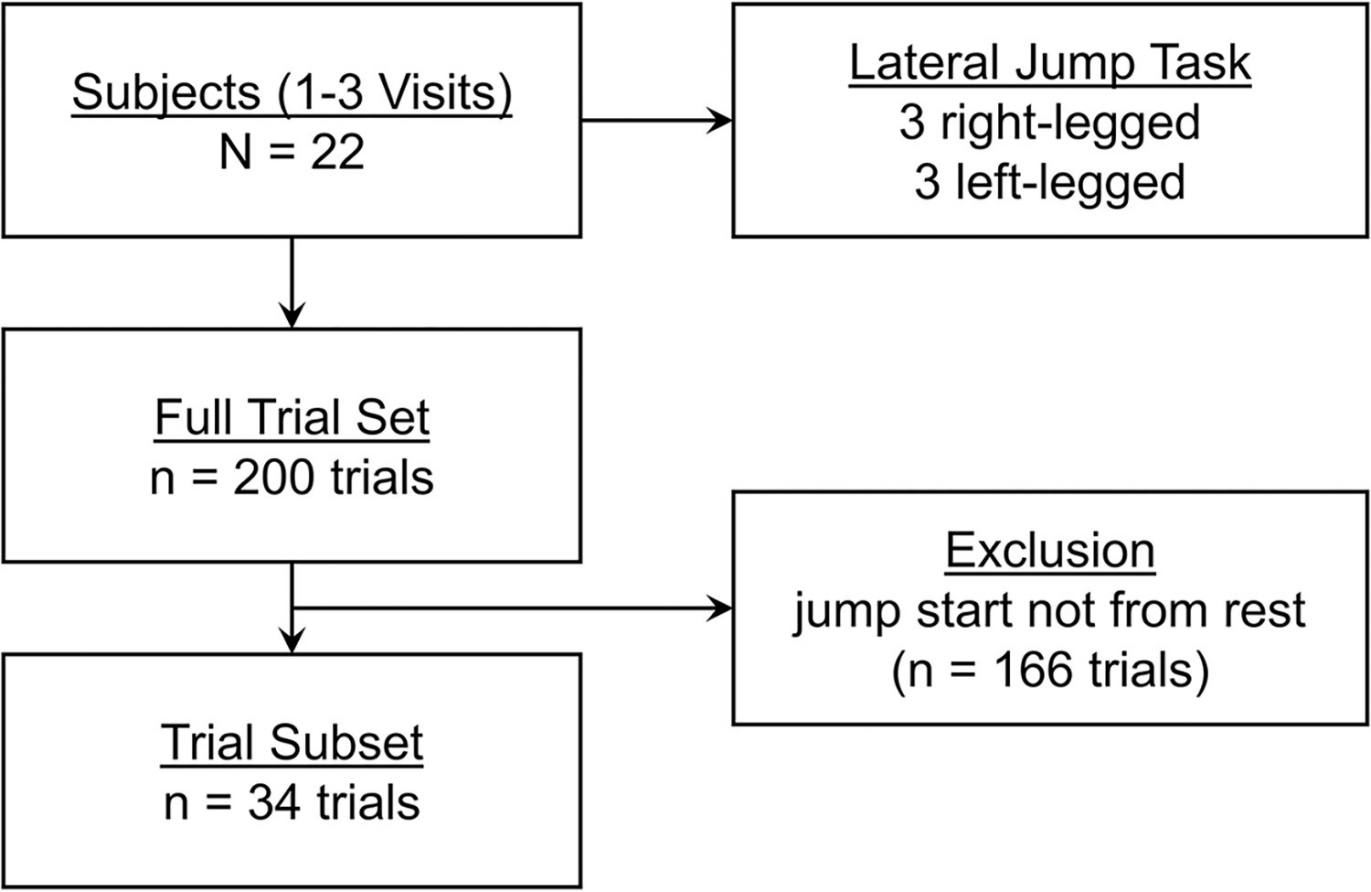
Flow chart of trial sets.

Computations of the various jump phase-specific variables were attempted for all lateral jump trials; however, the phase boundaries were not able to be accurately identified for several trials. The jump task protocol used for this study did not specify a period of rest before jumping. The integration of the force-time curve to derive velocity operates under the assumption that the jump motion begins with zero initial velocity, which means the subject is motionless or at rest. Although the impulse could still be computed for such trials, instantaneous velocity could not be determined without an initial velocity reference point and thus the phase boundaries, which are defined by a zero vertical velocity time point, could not be ascertained as well. Therefore, a post-testing inclusion criterion was established for exploratory purposes to identify trials in which the subject maintained a motionless period of rest in duration of at least 0.25 seconds before the onset of jump movement. ‘Rest’ was defined as a vertical ground reaction force within ± 5% of BW. 166 trials did not meet this criterion and thus were not further analyzed. A total of 34 trials met the established criteria and were included in the exploratory subset analysis. Fig 3 illustrates the breakdown of the full trial set and subsets.

For the 34-trial subset, the following additional independent variables were computed as by Laffaye, Wagner (6): absolute and relative average eccentric braking rate of force development (ECC-RFD) for all three force curves, absolute and relative average concentric force (CON-AVG) for all three force curves, eccentric time (ECC-T), total time (TIME), and eccentric to total time ratio (ECC-T:T). ECC-RFD is the average slope of the force-time curve for the eccentric braking phase. It was computed for each force-time curve as an absolute (N/s) and relative (N/kg‧s) value. CON-AVG is the average ground reaction force during the concentric phase. It was computed for each force-time curve as an absolute (N) and relative (N/kg) value. ECC-T (s) is the eccentric phase duration and TIME (s) is the total jump duration. ECC-T:T is the ratio of eccentric phase time to total time.

### Statistical analyses

Statistical analyses were conducted using Stata (Stata 8, Stata Corporation, College Station, TX). Descriptive statistics (means and standard deviations) were computed for all metrics in both trial sets. Two sets of pairwise correlations, one for the 200-trial set and one for the 34-trial subset, were used to assess the relationship between each of the force-plate derived predictor variables and the response variable, jump distance. A correlation coefficient of less than 0.1 was defined as negligible, 0.1 to 0.39 as weak, 0.4 to 0.69 as moderate, and greater than 0.7 as strong [36]. Three stepwise multiple regression models were fitted using the predictor variables and jump distance as the response variable. For the 200-trial set, one model was fitted using force predictor variables normalized to body mass and another was fitted using raw (unnormalized) force predictor variables. Only metrics that showed a significant pairwise correlation to jump distance were included in the models. A third model was fitted from the 34-trial subset, again using only predictor variables that showed a significant pairwise correlation to jump distance. An alpha level of 0.05 was selected *a priori* to determine if the predictor variables were significant, if predictor variables would be included in the regression models, and if the models were significant.

## Results

The means and standard deviations for each of the variables in the two trial sets can be seen in Table 1. The pairwise correlations between lateral jump distance and each predictor variable are in Table 2. In the full set of trials, lateral jump distance showed a significant positive correlation with pGRF_y_ and Relative pGRF_y_ and a significant negative correlation with pGRF_z_, Relative pGRF_z_, Relative pGRF_r_, and θ_r_. Each of these variables, however, displayed only a weak correlation. In the subset of trials (n = 34), lateral jump distance showed a significant positive correlation with Relative CON-AVG_y_ and TIME and a significant negative correlation with pGRF_r_, Relative pGRF_r_, Relative pGRF_z_, and θ_r_. Relative pGRF_z_, Relative pGRF_r_, and θ_r_ had a moderate correlation, whereas the others had a low correlation.

**Table 1:** Descriptive statistics (mean ± standard deviations) for the full trial set and trial subset.

Variable	Full Set (n = 200)		Subset (n = 34)	
Jump Distance	2.04	± 0.18	2.04	± 0.17
Peak Ground Reaction Force (N)				
Lateral	762	± 110	782	± 88
Vertical	1752	± 285	1824	± 198
Resultant	1893	± 296	1966	± 209
Relative Peak Ground Reaction Force (N/kg)				
Lateral	8.06	± 0.83	7.86	± 0.66
Vertical	18.50	± 1.89	18.32	± 1.38
Resultant	19.99	± 1.91	19.75	± 1.40
Ground Reaction Force Angle at pGRFr	67.5	± 2.8	67.9	± 1.9
Eccentric Braking RFD (N/s)				
Lateral		-	1624	± 407
Vertical		-	3009	± 1286
Resultant		-	3332	± 1354
Relative Eccentric Braking RFD (N/kg*s)				
Lateral		-	16.5	± 4.8
Vertical		-	30.5	± 13.9
Resultant		-	33.8	± 14.8
Average Concentric Force (N)				
Lateral		-	585	± 67
Vertical		-	1404	± 142
Resultant		-	1525	± 148
Relative Average Concentric Force (N/kg)				
Lateral		-	5.88	± 0.59
Vertical		-	14.1	± 0.77
Resultant		-	15.3	± 0.81
Eccentric Time (s)		-	0.608	± 0.123
Total Time (s)		-	0.862	± 0.131
Eccentric Time: Total Time Ratio		-	0.702	± 0.061

Abbreviations: RFD, rate of force development

**Table 2:** Pairwise correlations between the response variable (jump distance) and the predictor variables.

Variable	Full Set (n = 200)		Subset (n = 34)	
	R	p-value	R	p-value
Peak Ground Reaction Force (N)				
Lateral	0.2198	0.002	0.1813	0.305
Vertical	-0.1543	0.029	-0.2912	0.095
Resultant	-0.1143	0.107	-0.2467	0.016
Relative Peak Ground Reaction Force (N/kg)				
Lateral	0.3096	p<0.001	0.1789	0.311
Vertical	-0.2152	0.002	-0.4781	0.004
Resultant	-0.1585	0.025	-0.4343	0.010
Ground Reaction Force Angle at pGRFr	-0.3703	p<0.001	-0.4283	0.012
Eccentric Braking RFD (N/s)				
Lateral	-	-	0.0404	0.820
Vertical	-	-	-0.2159	0.220
Resultant	-	-	-0.1939	0.272
Relative Eccentric Braking RFD (N/kg*s)				
Lateral	-	-	0.0374	0.834
Vertical	-	-	-0.1912	0.279
Resultant	-	-	-0.1698	0.337
Average Concentric Force (N)				
Lateral	-	-	0.3334	0.054
Vertical	-	-	-0.1159	0.514
Resultant	-	-	-0.0402	0.822
Relative Average Concentric Force (N/kg)				
Lateral	-	-	0.3453	0.045
Vertical	-	-	-0.3094	0.075
Resultant	-	-	-0.1681	0.342
Eccentric Time (s)	-	-	0.2236	0.204
Total Time (s)	-	-	0.3555	0.039
Eccentric Time: Total Time Ratio	-	-	-0.1230	0.488

Abbreviations: pGRFr, resultant peak ground reaction force; RFD, rate of force development

Each significantly correlated predictor variable was inputted to a multiple linear regression model as seen in Tables 3–5. The final equation in Table 3 included pGRF_y_, pGRF_z_, and θ_r_ from the full cohort. This model accounts for 27.3% of the variation in lateral jump distance (p<0.001). The final equation in Table 4 included Relative pGRF_y_, Relative pGRF_z_, and θ_r_ from the full cohort. This model accounts for 21.4% of the variation in lateral jump distance (p<0.001). The final equation in Table 5 included Relative CON-AVG_y_ and Relative pGRF_r_. This model accounts for 55.2% of the variation in lateral jump distance in the sub-cohort.

**Table 3:** Multiple linear regression model predicting lateral jump distance; derived with full trial set, absolute variables.

Multiple Linear Regression Model					
Source	SS	df	MS	Observations	200
Model	1.7429	3	0.5810	F(3,196)	24.51
Residual	4.6450	196	0.0237	Prob > F	0.0000
Total	6.3879	199	0.0321	R^2^	0.2728
				Adjusted R^2^	0.2617
Predictor Variables	Coefficient		t	p-value	
pGRFy	0.0018		6.04	0.000	
pGRFz	-0.0007		-5.80	0.000	
Angle	0.0246		2.76	0.006	
Constant	0.1997		0.32	0.749	

Abbreviations: SS, sum of the squares; df, degrees of freedom; MS, mean squares; pGRFy, lateral peak ground reaction force; pGRFz, vertical peak ground reaction force

**Table 4:** Multiple linear regression model predicting lateral jump distance; derived with full trial set, relative variables.

Multiple Linear Regression Model					
Source	SS	df	MS	Observations	200
Model	1.3683	2	0.6842	F(2,197)	26.85
Residual	5.0196	197	0.0255	Prob > F	0.0000
Total	6.3879	199	0.0321	R^2^	0.2142
				Adjusted R^2^	0.2062
Predictor Variables	Coefficient		t	p-value	
Relative pGRFy	0.0939		6.49	0.000	
Relative pGRFz	-0.0347		-5.45	0.000	
Constant	1.9273		14.33	0.000	

Abbreviations: SS, sum of the squares; df, degrees of freedom; MS, mean squares; pGRFy, lateral peak ground reaction force; pGRFz, vertical peak ground reaction force

**Table 5:** Multiple linear regression model predicting lateral jump distance; derived with trial subset variables.

Multiple Linear Regression Model					
Source	SS	df	MS	Observations	34
Model	0.5361	2	0.2681	F(2, 31)	19.10
Residual	0.4351	31	0.0140	Prob > F	0.0000
Total	0.9712	33	0.0294	R^2^	0.552
				Adjusted R^2^	0.5231
Predictor Variables	Coefficient		t	p-value	
Relative CON-AVGy	0.1962		5.01	0.000	
Relative pGRFr	-0.0902		-5.47	0.000	
Constant	2.6651		8.77	0.000	

Abbreviations: SS, sum of the squares; df, degrees of freedom; MS, mean squares; CON-AVGy, lateral average concentric force; pGRFr, resultant peak ground reaction force

## Discussion

The purpose of this study was to identify three-dimensional force-plate derived predictors for jump distance in a lateral jump task. It was hypothesized that a regression model based on commonly measured force-time metrics during the jump would be able to significantly predict lateral jump distance. This hypothesis was partially supported because the three derived equations were able to significantly predict lateral jump distance, but not all predictor variables were included in the equations. These findings have implications regarding the use of multidirectional force plates and potential incorporation of a lateral jump task in basketball performance evaluations.

Both the lateral and vertical peak ground reaction forces, absolute and relative, were included in the two final models derived from the full trial set, thus suggesting increased lateral force production, in addition to vertical force production, is a pertinent mechanism to the lateral jump. This is supported by prior research that found the lateral jump involves unique leg power qualities from the vertical jump, as evidenced by a lack of shared variance between a vertical and lateral CMJ task [12]. Lateral movements exhibit a unique SSC from vertical movements [37] and utilize muscles such as the hip extensors that are not used when moving forward and/or vertically [24]. Meylan et al [13] similarly found that ground reaction force generation is different in the lateral jump versus the vertical jump; however, they did not find lateral peak ground reaction force to be a significant predictor of lateral jump distance, which contradicts our findings. This may be explained by differences in population, as the Division-I athletes in our study produced lateral peak lateral ground reaction forces of over 100 N greater than those of the club level athletes measured in Meylan, Nosaka [13].

The inclusion of lateral relative average concentric force in the trial subset model demonstrates the importance of lateral propulsion during the jump, but the absence of lateral eccentric rate of force development differs from findings in vertical CMJs that equally stress the contribution of muscular loading in performance [6]. This may be a result of differences between the muscular SSC in lateral and vertical movements [37]. Laffaye, Wagner, and Tombleson [6] observed a similar concentric phase relationship in a vertical CMJ in that relative average concentric force correlated moderately with jump height. Both the current study and Laffaye, Wagner [6] demonstrate maximizing the concentric muscular contractions respective to the direction of the jump lead to greater jump performance. However, the authors also found vertical eccentric rate of force development to positively correlate with jump height and thus stipulated that improving both metrics would benefit vertical jump performance. Although our results did not show significant correlations with eccentric rate of force development, prior literature has found eccentric phase metrics to corelate with lateral jump performance [14]. This discrepancy may be due to sometimes poor reliability in measuring eccentric rate of force development [11]. It is therefore still possible that increased lateral eccentric rate of force development would contribute to lateral jump performance.

Although the lateral force metric represented in the models correlated positively with distance, vertical peak ground reaction force (full trial set) and resultant relative peak ground reaction (trial subset), displayed negative correlations—a finding that appears contradictory but may be explained by the subject population: collegiate men’s basketball players. Vertical peak ground reaction forces are known to correlate with vertical jump height [13]. Therefore, the negative correlation is likely due to task technique, in which the participants tended to emphasize jumping high over jumping far. Since the vertical force outputs are typically about three to five times greater than the lateral force outputs, the relationships seen in the resultant force metrics are likely due to the same reason. There likely exists an ideal ratio between vertical and lateral force generation that optimizes jump distance. This is supported by the inclusion of peak ground reaction force angle in the final prediction models and its negative correlation with jump distance. Analyses of ideal launch angles in the track and field long jump found that the ideal jump angle is several degrees below what most jumpers are comfortable with and that the closer to 90° the angle became, jump distance decreased [38]. The determination of an optimal technique for the lateral jump task would likely correct these findings but would require more extensive modeling.

The findings of this study have implications for elite basketball musculoskeletal training and evaluation. The identification of lateral force variables in our predictive models suggests that lateral ground reaction force production, in addition to vertical force production, is a predictor of lateral jump distance. Prior studies have shown that performance in vertical and lateral tasks occur independently from one another [12] and therefore training for lateral performance improvements needs to be intentional. The lateral ground reaction force metrics identified as task predictors in this study–lateral peak ground reaction force and lateral average concentric force–can be used as a starting point for performance improvement monitoring. This also stresses the importance of utilizing force plates with three-dimensional measurement capabilities for evaluating multidirectional performance in basketball players. The computation of these metrics requires measurement of lateral ground reaction forces that are not measurable with commonly used vertical-only force plates. The jump technique utilized in the lateral task should also be considered when trying to evaluate an athlete’s neuromuscular capabilities. Our results showed a negative relationship with multiple vertical ground reaction force metrics, and although counterintuitive, may be explained by the varying degree of vertical force emphasis in each subject’s jump technique. Therefore, when performing the lateral jump task, athletes should be given ample opportunities to practice the movement and determine their most optimal form. This should better allow the task to isolate the evaluation of neuromuscular capability over modifiable technique.

This study is not without limitations. The study participants consisted of men’s varsity basketball players from a single institution and thus our results are likely not generalizable to all sports and athletic levels. Additionally, certain aspects of the lateral jump task itself may have confounded some of our findings. First, the task allowed an arm swing, which is often not allowed in a standard countermovement jump. Arm swings have been shown to significantly increase performance in lateral jumps [39]. As the arm swing is a learned skill, its inclusion likely benefited some participants more than others and thus reduced the task’s ability to access raw neuromuscular ability. Second, jump distance was measured as the point of takeoff to the landing point of the non-takeoff foot. This allowed for factors such as increased leg length and flexibility to potentially increase a participant’s jump distance without producing greater force outputs. Lastly, the jump motion did not start from a point of rest for many of the subjects and necessitated the development of the sub-cohort to be able to compute several of the force-time variables. The sample size of the sub-cohort was relatively small due to the established criteria. Despite its statistical significance, the sub-cohort regression model should be interpreted with caution. Subsequent studies should be conducted with a more controlled lateral jump task to identify stronger predictors. Further research should aim to identify training strategies that elicit specific improvements in the identified lateral jump distance predictor metrics. Investigation of which metrics are directly modifiable and how to improve each metric will ultimately help accomplish the goal of optimizing lateral performance.

### Practical application

The lateral jump task methodology outlined in this study can be beneficial to sports scientists and strength and conditioning practitioners. A lateral jump task may be worthwhile to incorporate in neuromuscular evaluations for basketball players. The use of three-dimensional force plates is recommended when doing so, as it will allow for the quantification of lateral forces during the jump motion. This study identified several predictor metrics, including relative CON-AVG_y_ and relative pGRF_r_, which practitioners can monitor before, during, and after basketball season to gauge neuromuscular ability and track improvement. It should be noted, however, that specific training interventions to directly modify the jump predictor metrics still need to be determined.

## Conclusions

Basketball is a multidirectional sport and therefore neuromuscular evaluations should be used to identify areas for improvements in all three dimensions. Several force plate-derived metrics were identified as significant predictors of lateral jump distance but a regression model with Relative CON-AVG_y_ and Relative pGRF_r_ explained a greater variability in performance (R^2^ = 0.55) compared to the other variables which were low, yet also significant. Our results suggest that lateral ground reaction forces can be used to evaluate lateral jump performance with the use of three-dimensional force plates. The lateral ground reaction force metrics identified as task predictors in this study can be used as a starting point for performance improvement monitoring. Interventions that directly modify the predictor metrics can be incorporated into basketball training regimens to optimize lateral performance.

## Supporting information

S1 File Force plate data file for lateral jumps. (XLSX)
